# Investigations into the relationship between feedback loops and functional importance of a signal transduction network based on Boolean network modeling

**DOI:** 10.1186/1471-2105-8-384

**Published:** 2007-10-15

**Authors:** Yung-Keun Kwon, Sun Shim Choi, Kwang-Hyun Cho

**Affiliations:** 1 Department of Bio and Brain Engineering and KI for the BioCentury, Korea Advanced Institute of Science and Technology, 335 Gwahangno, Yuseong-gu, Daejeon, 305-701, Republic of Korea; 2 College of Bioscience and Biotechnology and Institute of Bioscience and Biotechnology, Kangwon National University, Chuncheon-si, Gangwon-do, 200-701, Republic of Korea

## Abstract

**Background:**

A number of studies on biological networks have been carried out to unravel the topological characteristics that can explain the functional importance of network nodes. For instance, connectivity, clustering coefficient, and shortest path length were previously proposed for this purpose. However, there is still a pressing need to investigate another topological measure that can better describe the functional importance of network nodes. In this respect, we considered a feedback loop which is ubiquitously found in various biological networks.

**Results:**

We discovered that the number of feedback loops (NuFBL) is a crucial measure for evaluating the importance of a network node and verified this through a signal transduction network in the hippocampal CA1 neuron of mice as well as through generalized biological network models represented by Boolean networks. In particular, we observed that the proteins with a larger NuFBL are more likely to be essential and to evolve slowly in the hippocampal CA1 neuronal signal transduction network. Then, from extensive simulations based on the Boolean network models, we proved that a network node with the larger NuFBL is likely to be more important as the mutations of the initial state or the update rule of such a node made the network converge to a different attractor. These results led us to infer that such a strong positive correlation between the NuFBL and the importance of a network node might be an intrinsic principle of biological networks in view of network dynamics.

**Conclusion:**

The presented analysis on topological characteristics of biological networks showed that the number of feedback loops is positively correlated with the functional importance of network nodes. This result also suggests the existence of unknown feedback loops around functionally important nodes in biological networks.

## Background

Topological or structural analysis of biological networks can provide us with new insights into the design principle and the evolutionary mechanism of network molecules [1-4]. For instance, it has been widely accepted that biological networks have scale-free characteristics and a few highly connected network nodes (hubs) play pivotal roles in maintaining the global network structure [5]. Moreover, some other topological characteristics such as connectivity, clustering coefficient, and shortest path length have been proposed to explain the evolutionary rate and/or the lethality of network nodes. It has been shown that highly connected proteins in protein-protein interaction networks have a higher clustering coefficient and a smaller shortest path length. Consqeuntly, such proteins are more likely to be essential and evolve slowly [1,3,6-8]. There is however a pressing need to develop another topological measure that can better explain the relationship between network characteristics and biological importance of network nodes [1,9].

We note that feedback loops are ubiquitously found in various biological networks and play important roles in amplifying (positive feedback loop) or inhibiting (negative feedback loop) intracellular signals [10-15]. It has been suggested that such a feedback loop could be an important network motif [16-18]. Yet, it has not been fully investigated whether there exists a correlation between feedback loops and the functional importance of network nodes. Hence, we address this problem here and propose that the number of feedback loops (NuFBL) is a novel network measure characterizing such a functional importance of network nodes.

To prove our hypothesis, we use the random Boolean network models where directed links between nodes are randomly chosen. This random Boolean network model has been widely used to represent various biological networks and it has successfully captured some biological properties [19-23]. For instance, random Boolean network models were used to prove the properties of the yeast transcriptional network in that the network converges to a same stable state and it is robust against mutations of initial states [19]. They were also used to explain the remarkable robustness observed in genetic regulatory networks [20] and some properties of cell cycle networks such as stability along with genome size and the number of active genes along with the in-degree distribution [21] were also explained by Boolean network models. Previous studies adopt these random Boolean network models to prove that the global dynamics of the genetic regulatory network of HeLa cells are highly ordered [22] and the dynamics of various biological networks such as multi-stability and oscillations are related with positive or negative feedback loops [23]. These previous studies have validated usefulness of the random Boolean network models in analyzing the dynamical characteristics of biological networks.

## Results and discussion

### Correlation between the functional importance of network nodes and the NuFBL

#### The hippocampal CA1 neuronal signal transduction network

**Figure 1:**
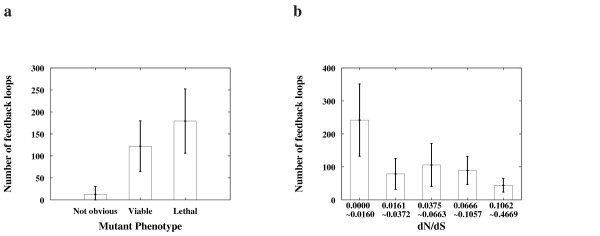
**Correlation between the functional importance of proteins and the NuFBL**. **(a) **The NuFBL's were plotted against the mutant phenotypes of the proteins in the network where proteins were classified according to the previous report [1]. **(b) **The NuFBL's were plotted against the evolutionary rate [1] (*dN*/*dS*) of proteins which were grouped into five different classes according to their evolutionary rates. For each protein group, the average and the confidence interval for 95% confidence level of the NuFBL are shown on the y-axis (see additional data file 4 for further details).

We considered the large signal transduction network of the hippocampal CA1 neuron of mice to examine the NuFBL as a new network measure [6]. We first confirmed the previous observation that proteins with a higher connectivity are more likely to be lethal and to have a slower evolutionary rate (data not shown). It has been considered that the lethal proteins are more essential than other proteins showing no obvious phenotype when deleted [1]. Also, it has been known that functionally important proteins are under a strong regulatory constraint resulting in relatively slow evolution [24,25]. Similarly, to examine whether the NuFBL of a protein is related to its functional importance, the NuFBL was plotted against the degree of phenotype and the evolutionary rate for grouped proteins as described in Methods. In Fig. 1, it was observed that more essential proteins (Fig. 1a) and more slowly evolving proteins (Fig. 1b) tend to have a larger NuFBL, which suggests that functionally important proteins in the signal transduction network are more likely to be regulated by many feedback loops. On the contrary, the nonessential proteins indicated by "Not obvious" phenotype group showed a very small NuFBL and they are less likely to be regulated by feedback loops. Note that most of the proteins except those with the slowest evolutionary rate have little difference in the NuFBL.

#### Boolean network models of biological networks

To further investigate whether the positive correlation between the NuFBL and the functional importance is an intrinsic principle of network dynamics, we performed extensive computer simulations for generalized biological network models represented by Boolean networks (see Methods). The importance of a node in the Boolean network model was defined as the probability with which either an initial state mutation or an update rule mutation of the node makes the network converge to a new attractor. In Boolean network models, a state trajectory starts from an initial state and eventually converges to either a fixed-point or a limit-cycle attractor. So, these attractors represent diverse behaviors of biological networks such as multistability, homeostasis, and oscillation [26-28]. For instance, in the regulatory network of inducing phenotype variations in bacteria, some epigenetic traits are represented by multiple fixed-point attractors [29]. In addition, mitogen-activated protein kinase cascades in animal cells [26,27] and cell cycle regulatory circuits in Xenopus and Saccharomyces cerevisiae [28,30] are known to produce multistable attractors. On the other hand, the transcriptional network of mRNAs for Notch signaling molecules shows the oscillation with a 2-h cycle by hes1 transcription [31] corresponding to a limit-cycle attractor. ¿From these examples, we can find that attractors represent essential dynamics of biological networks. Therefore, converging to a different attractor by some mutations at a node means that the node has a significant role in the network. This concept has been widely used in a number of previous studies based on computational approaches [32-35].

**Figure 2:**
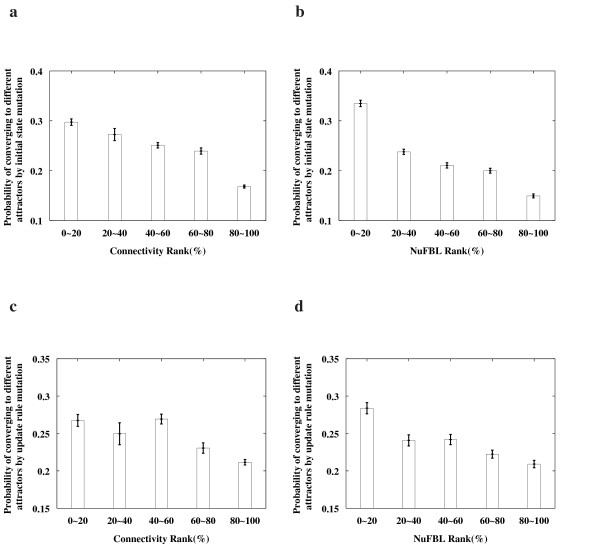
**Correlation of connectivity and the NuFBL to the functional importance in Boolean networks**. **(a) **Correlation between connectivity and the functional importance of network nodes with respect to initial state mutations. **(b) **Correlation between the NuFBL and the functional importance of network nodes with respect to initial state mutations. **(c) **Correlation between connectivity and the functional importance of network nodes with respect to update rule mutations. **(d) **Correlation between the NuFBL and the functional importance of network nodes with respect to update rule mutations. In each figure, all nodes were classified into five groups according to their connectivity or NuFBL ranks. All the results represent the average over randomly generated 2000 Boolean networks with |*V*| = 14 and |*A*| = 19. For each group, the average and the confidence interval for 95% confidence level of the functional importance are shown on the y-axis. Here, the functional importance of a network node is defined by the probability with which the network converges to a different attractor when the value of the node is mutated. For other Boolean networks with different |*V*| and |*A*|, we also obtained similar results (see additional data file 1).

Fig. 2 shows the results of the Boolean networks with |*V*| = 14 and |*A*| = 19. It turns out that the network nodes with a higher connectivity or NuFBL are more important, which is consistent with the observation in the above neuronal signal transduction network. And, we observed the same result for networks with different sizes (see additional data file 1). Moreover, we found that the NuFBL is a better network measure than the connectivity in evaluating the functional importance of a network node.

In addition to the NuFBL, we can think of another measure that represents the particular characteristics of feedback loops. For instance, we have investigated the relationship between the length of feedback loops at a node and its functional importance which is defined in the same way as in Fig. 2. In this case, the nodes with relatively longer or shorter loop lengths were functionally less important while the nodes with medium loop lengths were more important (see additional data file 2 for details). So, the length of feedback loops can be considered as another measure, but it is no longer linearly correlated with the functional importance unlike the NuFBL.

### Comparison of the NuFBL and the connectivity

#### Correlation between the NuFBL and the connectivity in the neuronal signal transduction network

**Figure 3:**
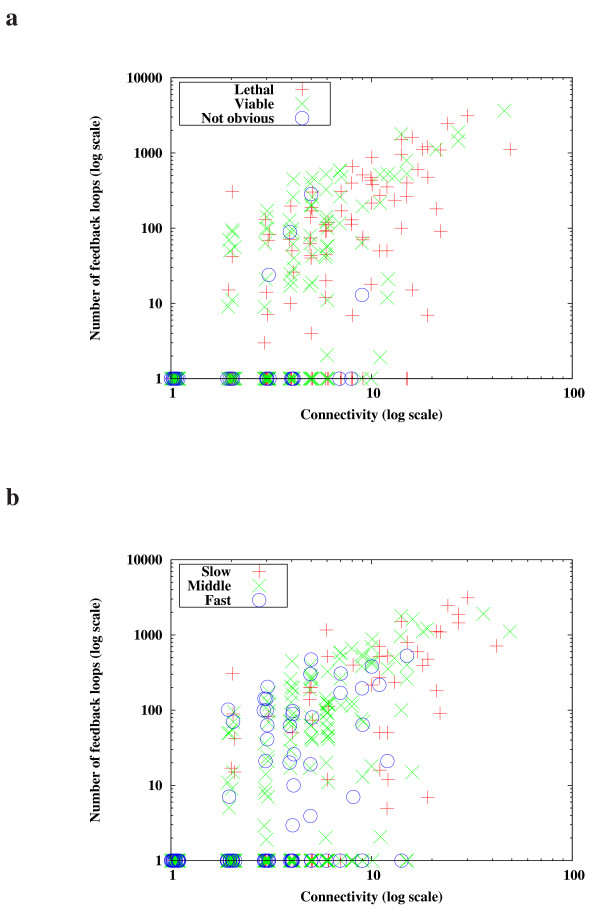
**Distribution of proteins with respect to connectivity and the NuFBL**. **(a) **Proteins were classified into "Lethal", "Viable", and "Not obvious", respectively, according to their mutant phenotypes. **(b) **Proteins were classified into "Slow", "Middle", and "Fast", respectively, according to their evolutionary rates (see additional data file 3 for further details).

We compared the NuFBL and the connectivity as a measure of network characteristics. As shown in Fig. 3, it was observed that there is a strong positive correlation between the connectivity and the NuFBL (the correlation coefficient is 0.73). Interestingly, the positive correlation was relatively stronger for the lethal and slowly-evolving proteins, which have a high connectivity and a large NuFBL (red plus sign points in Fig. 3a, b). On the contrary, there was only a weak correlation for the proteins of a non-lethal group or a rapidly evolving group (blue circle points in Fig. 3a, b). The correlation coefficient of 152 proteins whose connectivity ranged from 5 to 9 was only 0.14.

#### Classification of proteins in the CA1 neuronal signal transduction network

To probe the distribution of proteins, we classified the proteins into four different groups (see Methods): "no feedback loop & low connectivity", "no feedback loop & high connectivity", "feedback loop & low connectivity", and "feedback loop & high connectivity" (Table 1). The functional importance (R) estimated by the lethal mutant phenotype or slow evolutionary rate was significantly higher for the "feedback loop & high connectivity" group. Note that the connectivity or the NuFBL alone was not enough to discern all the different network characteristics.

**Table 1:** Classification of proteins and their functional importance in the hippocampal CA1 neuronal signal transduction network

The functional importance with respect to mutant phenotypes									
	No feedback loop			Feedback loop			Total		
	
	*N*	*U*	*R*	*N*	*U*	*R*	*N*	*U*	*R*
Low connectivity	49	142	34.5%	9	24	37.5%	58	166	34.9
High connectivity	19	55	34.5%	60	118	50.8%	79	173	45.7%

Total	68	197	34.5%	69	142	48.6%	137	339	40.4%
									
The functional importance with respect to evolutionary rates									

	No feedback loop			Feedback loop			Total		
	
	*N*	*U*	*R*	*N*	*U*	*R*	*N*	*U*	*R*

Low connectivity	36	208	17.3%	6	40	15.0%	42	248	16.9%
High connectivity	10	71	14.1%	37	136	27.2%	47	207	22.7%

Total	46	279	16.5%	43	176	24.4%	89	455	19.6%

*U *: The number of proteins belonging to the corresponding class.

*N *: The number of important proteins with either a "Lethal" phenotype or a "Slow" evolutionary rate.

*R *: *N*/*U *× 100(%).

**Figure 4:**
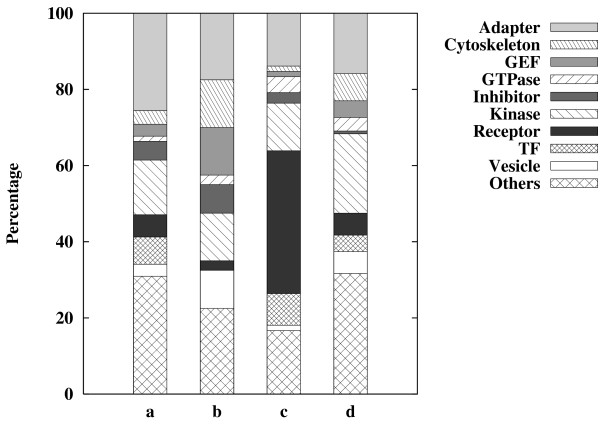
**Classification of proteins according to their function in the hippocampal CA1 neuronal signal transduction network and the proportion of each classified group**. The proteins were classified into four groups: **(a) **"no feedback loop & low connectivity" group, **(b) **"no feedback loop & high connectivity" group, **(c) **"feedback loop & low connectivity" group, and **(d) **"feedback loop & high connectivity" group. For each group, the proportion of proteins classified according to their functions is specified.

We analyzed the distinct features of the proteins in the four groups with respect to their functional roles (Fig. 4). Interestingly, we found that receptor proteins were enriched in the "high connectivity & no feedback loop" group (Fig. 4c) and that downstream kinases and proteins from receptors were enriched in the "high connectivity & feedback loop" group (Fig. 4d). These suggest that the downstream proteins from receptors in the signal transduction network are primarily responsible for intensification of signals and therefore feedback regulations are required for the amplification and control of signals [36,37].

#### Classification of proteins in the computational networks

By using simulations based on the Boolean network models, we further investigated the relationship between the connectivity and the NuFBL. The whole network nodes were classified into four groups as in Table 1, and the simulations confirmed that the connectivity is positively correlated with the NuFBL with respect to the functional importance of network nodes (Table 2). This was verified through other Boolean networks with different sizes (see additional data file 3). In particular, we note that the nodes involved with no feedback loop present comparatively low functional importance on average. This implies that if a protein is relatively important among the "no feedback loop" group, it is likely for us to discover a new feedback loop around this protein.

**Table 2:** Classification of network nodes and their functional importance in generalized Boolean network models

Boolean networks with |*V*| = 14 and |*A*| = 19 (initial state mutation)						
	No feedback loop		Feedback loop		Total	
	
	*U*	*E*(*L*)	*U*	*E*(*L*)	*U*	*E*(*L*)

Low connectivity	4281	0.0263 (0.00183)	9592	0.2317 (0.00615)	13873	0.1683 (0.00457)
High connectivity	1246	0.0426 (0.00492)	12881	0.2806 (0.00564)	14127	0.2596 (0.00528)

Total	5527	0.0300 (0.00181)	22473	0.2597 (0.00418)	28000	0.2144 (0.00354)
						
Boolean networks with |*V*| = 14 and |*A*| = 19 (update rule mutation)						

	No feedback loop		Feedback loop		Total	
	
	*U*	*E*(*L*)	*U*	*E*(*L*)	*U*	*E*(*L*)

Low connectivity	4379	0.1858 (0.00779)	9465	0.2459 (0.00579)	13844	0.2268 (0.00468)
High connectivity	1269	0.2196 (0.01539)	12887	0.2800 (0.00515)	14156	0.2745 (0.00490)

Total	5648	0.1934 (0.00697)	22352	0.2655 (0.00386)	28000	0.2510 (0.00340)

*U *: The number of proteins belonging to the corresponding class.

*E *: The average value of the functional importance with respect to either initial update mutations or rule update mutations.

*L *: The confidence interval for 95% confidence level.

## Conclusion

We propose the NuFBL as a new network measure that can characterize the functional importance of network nodes. We have shown that the NuFBL is positively correlated with the connectivity in measuring network characteristics, and the network nodes with a higher NuFBL and a higher connectivity are more essential (lethal) and evolve slowly. Through extensive computational simulations, we found that the positive correlation between the NuFBL and the functional importance is an intrinsic property of network dynamics.

Unfortunately, at present, there are few large-scale biological networks harboring the information about feedback loops. A future study will therefore include a verification of the presented results in many other kinds of real biological networks. As another future study, we need to investigate the characteristics of feedback loops that can help us to predict the functional importance of network nodes from other aspects of the data. Such characteristics include timing of expression, the number of members in the loop, and the integrative sign of multiple interactions.

## Methods

### Connectivity, feedback loop, loop length, and the number of feedback loops (NuFBL)

Given a network composed of a set of nodes and a set of links between the nodes, the connectivity of a node is defined as the number of links connected to the node. A feedback loop means a closed simple cycle where nodes are not revisited except the starting and ending nodes. For instance, *v*_0 _→ *v*_1 _→ *v*_2 _→ ⋯ → *v*_*L *-1 _→ *v*_*L *_is a feedback loop of length *L*(≥ 1) if there are links from *v*_*i*-1 _to *v*_*i *_(*i *= 1, 2,..., *L*) with *v*_0 _= *v*_*N *_and *v*_*j *_≠ *v*_*k *_for *j*, *k *∈ {0, 1,..., *L *- 1}. The NuFBL of a node *v *denotes the number of different feedback loops starting from *v*.

### Analysis of the hippocampal CA1 neuronal signal transduction network

We considered all 545 proteins and their 1258 interactions in the signal transduction network of the hippocampal CA1 neuron of mice [6]. Following the previous study [1], proteins were grouped together according to their lethality and evolutionary rates. As it is difficult to enumerate all possible feedback loops in such a large network, we considered only the feedback loops whose length (i.e., the number of links comprising a feedback loop) is less than or equal to 10. Important proteins are defined as those with "lethal" phenotypes and these are illustrated in the upper of Table 1. 20% of the most slowly-evolving proteins are illustrated in the lower of Table 1.

### Analysis of generalized biological network models represented by Boolean networks

Boolean network models composed of a set of Boolean variables and regulatory relationships between the variables have been widely used as a useful tool for investigating the complex dynamics of various biological networks [38,39]. In spite of their structural simplicity, Boolean networks can represent a variety of complex behaviors [23] and share many features with other continuous models [40,41]. We employed such a Boolean network model and described biological networks by a directed graph, *G *= (*V*, *A*) where *V *is a set of Boolean variables and *A *is a set of ordered pairs of the variables, called directed links (|*V*| and |*A*| denote the numbers of nodes and links, respectively). Each *v*_*i *_∈ *V *has the value of 1 ("on") or 0 ("off"). A directed link (*v*_*i*_, *v*_*j*_) has a positive ("activating") or negative ("inhibiting") relationship from *v*_*i *_to *v*_*j*_. The value of each variable *v*_*i *_at time *t *+ 1 is determined by the values of *k*_*i *_other variables vi1,vi2,⋯,viki
 MathType@MTEF@5@5@+=feaafiart1ev1aaatCvAUfKttLearuWrP9MDH5MBPbIqV92AaeXatLxBI9gBaebbnrfifHhDYfgasaacH8akY=wiFfYdH8Gipec8Eeeu0xXdbba9frFj0=OqFfea0dXdd9vqai=hGuQ8kuc9pgc9s8qqaq=dirpe0xb9q8qiLsFr0=vr0=vr0dc8meaabaqaciaacaGaaeqabaqabeGadaaakeaacqWG2bGDdaWgaaWcbaGaemyAaK2aaSbaaWqaaiabigdaXaqabaaaleqaaOGaeiilaWIaemODay3aaSbaaSqaaiabdMgaPnaaBaaameaacqaIYaGmaeqaaaWcbeaakiabcYcaSiabl+UimjabcYcaSiabdAha2naaBaaaleaacqWGPbqAdaWgaaadbaGaem4AaS2aaSbaaeaacqWGPbqAaeqaaaqabaaaleqaaaaa@3FA7@ having a link to *v*_*i *_at time *t *through a Boolean function fi:{0,1}ki→{0,1}
 MathType@MTEF@5@5@+=feaafiart1ev1aaatCvAUfKttLearuWrP9MDH5MBPbIqV92AaeXatLxBI9gBaebbnrfifHhDYfgasaacH8akY=wiFfYdH8Gipec8Eeeu0xXdbba9frFj0=OqFfea0dXdd9vqai=hGuQ8kuc9pgc9s8qqaq=dirpe0xb9q8qiLsFr0=vr0=vr0dc8meaabaqaciaacaGaaeqabaqabeGadaaakeaacqWGMbGzdaWgaaWcbaGaemyAaKgabeaakiabcQda6iabcUha7jabicdaWiabcYcaSiabigdaXiabc2ha9naaCaaaleqabaGaem4AaS2aaSbaaWqaaiabdMgaPbqabaaaaOGaeyOKH4Qaei4EaSNaeGimaaJaeiilaWIaeGymaeJaeiyFa0haaa@4115@. Hence, we can represent the update rule as *v*_*i*_(*t *+ 1) = fi(vi1(t),vi2(t),⋯,viki(t))
 MathType@MTEF@5@5@+=feaafiart1ev1aaatCvAUfKttLearuWrP9MDH5MBPbIqV92AaeXatLxBI9gBaebbnrfifHhDYfgasaacH8akY=wiFfYdH8Gipec8Eeeu0xXdbba9frFj0=OqFfea0dXdd9vqai=hGuQ8kuc9pgc9s8qqaq=dirpe0xb9q8qiLsFr0=vr0=vr0dc8meaabaqaciaacaGaaeqabaqabeGadaaakeaacqWGMbGzdaWgaaWcbaGaemyAaKgabeaakiabcIcaOiabdAha2naaBaaaleaacqWGPbqAdaWgaaadbaGaeGymaedabeaaaSqabaGccqGGOaakcqWG0baDcqGGPaqkcqGGSaalcqWG2bGDdaWgaaWcbaGaemyAaK2aaSbaaWqaaiabikdaYaqabaaaleqaaOGaeiikaGIaemiDaqNaeiykaKIaeiilaWIaeS47IWKaeiilaWIaemODay3aaSbaaSqaaiabdMgaPnaaBaaameaacqWGRbWAdaWgaaqaaiabdMgaPbqabaaabeaaaSqabaGccqGGOaakcqWG0baDcqGGPaqkcqGGPaqkaaa@4DB2@ where we randomly use either a logical conjunction or disjunction for all the signed relationships in *f*_*i*_. For instance, if a Boolean variable *v *has a positive relationship from *v*_1 _and a negative relationship from *v*_2_, the conjunction and disjunction update rules are *v*(*t *+ 1) = *v*_1_(*t*) ∧ v2¯
 MathType@MTEF@5@5@+=feaafiart1ev1aaatCvAUfKttLearuWrP9MDH5MBPbIqV92AaeXatLxBI9gBaebbnrfifHhDYfgasaacH8akY=wiFfYdH8Gipec8Eeeu0xXdbba9frFj0=OqFfea0dXdd9vqai=hGuQ8kuc9pgc9s8qqaq=dirpe0xb9q8qiLsFr0=vr0=vr0dc8meaabaqaciaacaGaaeqabaqabeGadaaakeaadaqdaaqaaiabdAha2naaBaaaleaacqaIYaGmaeqaaaaaaaa@2F50@(*t*)and *v*(*t *+ 1) = *v*_1_(*t*) ∨ v2¯
 MathType@MTEF@5@5@+=feaafiart1ev1aaatCvAUfKttLearuWrP9MDH5MBPbIqV92AaeXatLxBI9gBaebbnrfifHhDYfgasaacH8akY=wiFfYdH8Gipec8Eeeu0xXdbba9frFj0=OqFfea0dXdd9vqai=hGuQ8kuc9pgc9s8qqaq=dirpe0xb9q8qiLsFr0=vr0=vr0dc8meaabaqaciaacaGaaeqabaqabeGadaaakeaadaqdaaqaaiabdAha2naaBaaaleaacqaIYaGmaeqaaaaaaaa@2F50@(*t*), respectively. We defined the functional importance of a node in Boolean networks as follows: Given a network with *N *Boolean variables, a *state *denotes a vector consisting of *N *Boolean variables; there are 2^*N *^states in total. Each state makes a transition to another state through the Boolean update function. We constructed a *state transition network *that describes the transition of all the states. For a network node *v*, its functional importance can be considered in two ways. One is the functional importance with respect to initial state mutations. It is defined as the probability with which two state trajectories starting from *s *and *s' *converge to different attractors for all 2^*N*-1 ^pairs of states *s *and *s' *having different values only at *v*. The initial state mutation corresponds to the abnormal state (or malfunctioning) of a protein or gene caused by mutations. The other is the functional importance with respect to the update rule mutations. It is defined as the probability with which two state trajectories starting from a same state converge to different attractors where one of the two trajectories is obtained without the update rule mutation and the other is obtained by an error in updating the value of *v *with a probability 0.2. The update rule mutation corresponds to the change of relationships between nodes by removal or addition of links.

## List of abbreviations

NuFBL: Number of feedback loops

## Authors' contributions

YKK conceived of the study, wrote the program code and drafted the manuscript. SSC and KHC were involved in drafting the manuscript and revising it critically. KHC guided the study and coordinated the project. All authors read and approved the final manuscript.

## Supplementary Material

Additional file 1 **The figure shows the correlation of connectivity and the NuFBL to the functional importance in Boolean networks**. **(a) **Correlation between connectivity and the functional importance of nodes with respect to initial state mutations in Boolean networks with |*V*| = 10 and |*A*| = 14. **(b) **Correlation between the NuFBL and the functional importance of nodes with respect to initial state mutations in Boolean networks with |*V*| = 10 and |*A*| = 14. **(c) **Correlation between connectivity and the functional importance of nodes with respect to update rule mutations in Boolean networks with |*V*| = 10 and |*A*| = 14. **(d) **Correlation between the NuFBL and the functional importance of nodes with respect to update rule mutations in Boolean networks with |*V*| = 10 and |*A*| = 14. **(e) **Correlation between connectivity and the functional importance of nodes with respect to initial state mutations in Boolean networks with |*V*| = 12 and |*A*| = 16. **(f) **Correlation between the NuFBL and the functional importance of nodes with respect to initial state mutations in Boolean networks with |*V*| = 12 and |*A*| = 16. **(g) **Correlation between connectivity and the functional importance of nodes with respect to update rule mutations in Boolean networks with |*V*| = 12 and |*A*| = 16. **(h) **Correlation between the NuFBL and the functional importance of nodes with respect to update rule mutations in Boolean networks with |*V*| = 12 and |*A*| = 16. All the results are the average over randomly generated 2000 Boolean networks. For each group, the average and the confidence interval for 95% confidence level of the functional importance are shown on the y-axis. Click here for file

Additional file 2 **The figure shows the correlation between the length of feedback loops and the functional importance in Boolean networks**. **(a) **Correlation between the length of feedback loops and the functional importance of nodes with respect to initial state mutations in Boolean networks with |*V*| = 14 and |*A*| = 19. **(b) **Correlation between the length of feedback loops and the functional importance of nodes with respect to update rule mutations in Boolean networks with |*V*| = 14 and |*A*| = 19. All the results are the average over randomly generated 2000 Boolean networks. In each figure, all nodes were classified into five groups according to the average length of feedback loops that are involved at each node. For each group, the average and the confidence interval for 95% confidence level of the functional importance are shown on the y-axis. Click here for file

Additional file 3 **The table shows classification of network nodes with respect to their connectivity and feedback loops in generalized biological networks represented by Boolean models**. The first and the second tables show the results with respect to initial state mutations and update rule mutations, respectively, in Boolean networks with |*V*| = 10 and |*A*| = 14. The third and fourth tables show the results with respect to initial state mutations and update rule mutations, respectively, in Boolean networks with |*V*| = 12 and |*A*| = 16. Click here for file

Additional file 4 **The table shows classification of proteins in the hippocampal CA1 neuronal signal transduction network**. The upper and the lower tables are the results with respect to classification of proteins according to their mutant phenotypes and classification of proteins according to their evolutionary rates, respectively. Click here for file
