# Supplementary figures and images for: Investigations into the relationship between feedback loops and functional importance of a signal transduction network based on Boolean network modeling
Source: BMC Bioinformatics. 2007 Oct 15;8:384. doi: 10.1186/1471-2105-8-384 (PMC2100072; doi:10.1186/1471-2105-8-384)

## Additional Data File 1

**a**

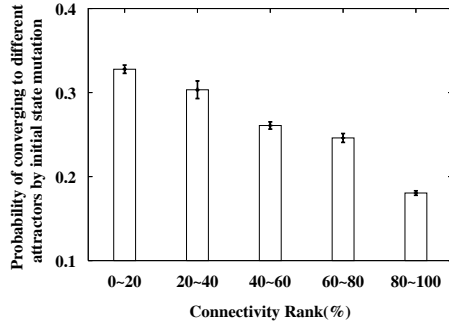

**b**

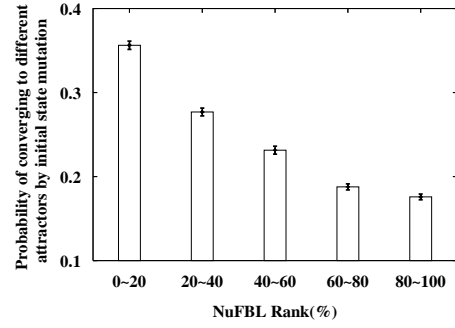

**c**

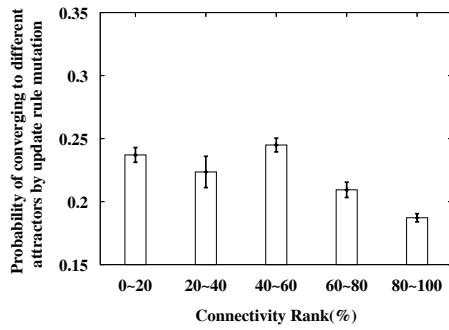

**d**

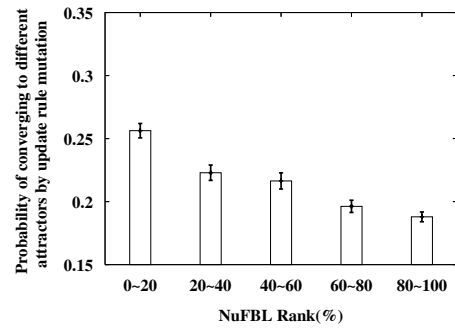

**e**

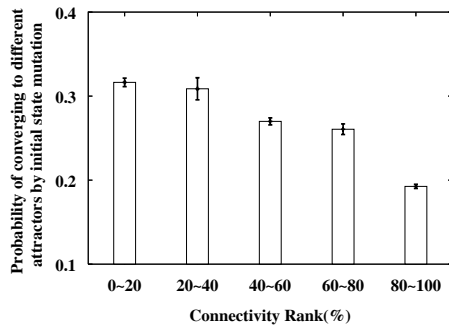

**f**

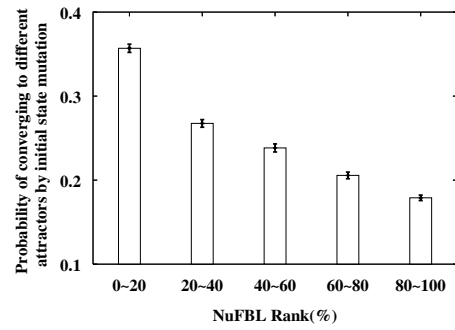

**g**

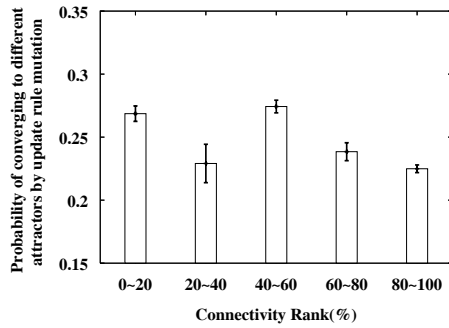

**h**

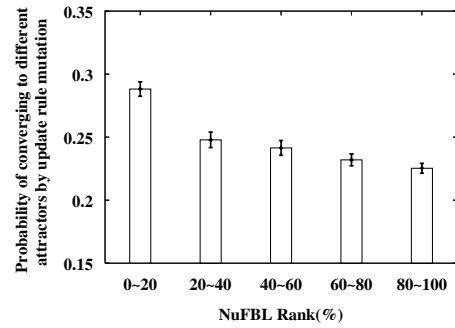

Supplement: Additional file 1 — The figure shows the correlation of connectivity and the NuFBL to the functional importance in Boolean networks. (a) Correlation between connectivity and the functional importance of nodes with respect to initial state mutations in Boolean networks with |V| = 10 and |A| = 14. (b) Correlation between the NuFBL and the functional importance of nodes with respect to initial state mutations in Boolean networks with |V| = 10 and |A| = 14. (c) Correlation between connectivity and the functional importance of nodes with respect to update rule mutations in Boolean networks with |V| = 10 and |A| = 14. (d) Correlation between the NuFBL and the functional importance of nodes with respect to update rule mutations in Boolean networks with |V| = 10 and |A| = 14. (e) Correlation between connectivity and the functional importance of nodes with respect to initial state mutations in Boolean networks with |V| = 12 and |A| = 16. (f) Correlation between the NuFBL and the functional importance of nodes with respect to initial state mutations in Boolean networks with |V| = 12 and |A| = 16. (g) Correlation between connectivity and the functional importance of nodes with respect to update rule mutations in Boolean networks with |V| = 12 and |A| = 16. (h) Correlation between the NuFBL and the functional importance of nodes with respect to update rule mutations in Boolean networks with |V| = 12 and |A| = 16. All the results are the average over randomly generated 2000 Boolean networks. For each group, the average and the confidence interval for 95% confidence level of the functional importance are shown on the y-axis. [file 1471-2105-8-384-S1.pdf]

## Additional Data File 2

a

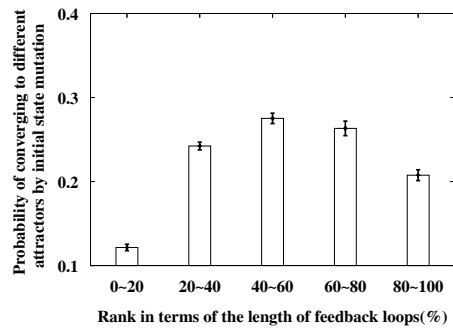

b

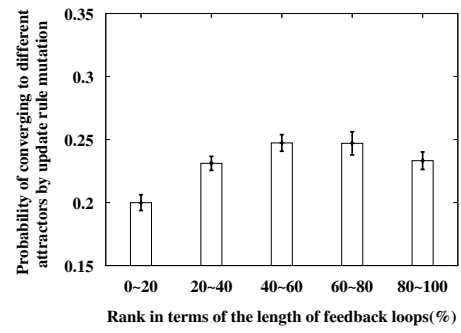

Supplement: Additional file 2 — The figure shows the correlation between the length of feedback loops and the functional importance in Boolean networks. (a) Correlation between the length of feedback loops and the functional importance of nodes with respect to initial state mutations in Boolean networks with |V| = 14 and |A| = 19. (b) Correlation between the length of feedback loops and the functional importance of nodes with respect to update rule mutations in Boolean networks with |V| = 14 and |A| = 19. All the results are the average over randomly generated 2000 Boolean networks. In each figure, all nodes were classified into five groups according to the average length of feedback loops that are involved at each node. For each group, the average and the confidence interval for 95% confidence level of the functional importance are shown on the y-axis. [file 1471-2105-8-384-S2.pdf]
