# Supplementary material for: Investigations into the relationship between feedback loops and functional importance of a signal transduction network based on Boolean network modeling
Source: BMC Bioinformatics. 2007 Oct 15;8:384. doi: 10.1186/1471-2105-8-384 (PMC2100072; doi:10.1186/1471-2105-8-384)
Supplement: Additional file 3 — The table shows classification of network nodes with respect to their connectivity and feedback loops in generalized biological networks represented by Boolean models. The first and the second tables show the results with respect to initial state mutations and update rule mutations, respectively, in Boolean networks with |V| = 10 and |A| = 14. The third and fourth tables show the results with respect to initial state mutations and update rule mutations, respectively, in Boolean networks with |V| = 12 and |A| = 16. [file 1471-2105-8-384-S3.pdf]

Additional Data File 3

Boolean networks with $|V| = 10$ and $|A| = 14$ (initial state mutation)

	No feedback loop		Feedback loop		Total	
	U	$E(L)$	U	$E(L)$	U	$E(L)$
Low connectivity	3048	0.0329 (0.00238)	6364	0.2632 (0.00786)	9412	0.1886 (0.00580)
High connectivity	641	0.0525 (0.00746)	9947	0.3086 (0.00663)	10588	0.2931 (0.00636)
Total	3689	0.0363 (0.00237)	16311	0.2909 (0.00509)	20000	0.2439 (0.00439)

Boolean networks with $|V| = 10$ and $|A| = 14$ (update rule mutation)

	No feedback loop		Feedback loop		Total	
	U	$E(L)$	U	$E(L)$	U	$E(L)$
Low connectivity	2978	0.1534 (0.00894)	6387	0.2221 (0.00660)	9365	0.2003 (0.00536)
High connectivity	735	0.1723 (0.01801)	9900	0.2532 (0.00558)	10635	0.2476 (0.00535)
Total	3713	0.1572 (0.00801)	16287	0.2410 (0.00427)	20000	0.2254 (0.00381)

Boolean networks with $|V| = 12$ and $|A| = 16$ (initial state mutation)

	No feedback loop		Feedback loop		Total	
	U	$E(L)$	U	$E(L)$	U	$E(L)$
Low connectivity	3794	0.0331 (0.00214)	8421	0.2604 (0.00678)	12215	0.1898 (0.00507)
High connectivity	763	0.0577 (0.00753)	11022	0.3039 (0.00618)	11785	0.2880 (0.00591)
Total	4557	0.0372 (0.00220)	19443	0.2851 (0.00458)	24000	0.2380 (0.00393)

Boolean networks with $|V| = 12$ and $|A| = 16$ (update rule mutation)

	No feedback loop		Feedback loop		Total	
	U	$E(L)$	U	$E(L)$	U	$E(L)$
Low connectivity	3664	0.1947 (0.00877)	8569	0.2626 (0.00606)	12233	0.2423 (0.00502)
High connectivity	750	0.1979 (0.01874)	11017	0.2917 (0.00552)	11767	0.2857 (0.00532)
Total	4414	0.1953 (0.00795)	19586	0.2790 (0.00409)	24000	0.2636 (0.00367)

U : The number of proteins belonging to the corresponding class.
E : The average value of the functional importance with respect to either initial update mutations or rule update mutations.
L : The confidence interval for 95% confidence level.
