# Supplementary material for: Investigations into the relationship between feedback loops and functional importance of a signal transduction network based on Boolean network modeling
Source: BMC Bioinformatics. 2007 Oct 15;8:384. doi: 10.1186/1471-2105-8-384 (PMC2100072; doi:10.1186/1471-2105-8-384)
Supplement: Additional file 4 — The table shows classification of proteins in the hippocampal CA1 neuronal signal transduction network. The upper and the lower tables are the results with respect to classification of proteins according to their mutant phenotypes and classification of proteins according to their evolutionary rates, respectively. [file 1471-2105-8-384-S4.pdf]

Additional Data File 4

Classification of proteins according to their mutant phenotypes

	Number of proteins	Connectivity	NuFBL
Lethal	137	6.657[a] (1.137[b])	179.36 (73.08)
Viable	170	4.618 (0.773)	122.17 (57.57)
No obvious	32	2.875 (0.713)	12.66 (17.63)

Classification of proteins according to their evolutionary rates

	Range of evolutionary rate	Number of proteins	Connectivity	NuFBL
Slow	0.0000 ∼ 0.0160	91	7.275 (1.636)	241.98 (109.53)
Middle	0.0161 ∼ 0.0372	91	4.462 (1.173)	78.68 (46.76)
	0.0375 ∼ 0.0663	91	4.736 (1.014)	105.82 (65.06)
	0.0666 ∼ 0.1057	91	4.088 (0.684)	89.27 (42.46)
Fast	0.1062 ∼ 0.4669	91	3.538 (0.602)	44.26 (20.60)

[a] The average.
[b] The confidence interval for 95% confidence level.
